# Integrated methylome–transcriptome profiling reveals epigenetic regulation of immune activation pathways and *CSN3*-associated lactation repression in bovine subclinical mastitis

**DOI:** 10.1186/s40104-026-01400-3

**Published:** 2026-05-10

**Authors:** Shraddha Dwivedi, Amit Kumar, Ujjwal Kumar De, Anuj Chauhan, Ravi Kant Agrawal, Shivani Khanna, Amritanshu Upadhyay, Ayushi Singh, Prem Chand Devatwal, Triveni Dutt

**Affiliations:** 1 Division of Animal Genetics, ICAR – Indian Veterinary Research Institute, Izatnagar, Bareilly, UP 243122 India; 2 Division of Medicine, Indian Veterinary Research Institute, Izatnagar, Bareilly, UP 243122 India; 3 Division of Livestock Production and Management, Indian Veterinary Research Institute, Izatnagar, Bareilly, UP 243122 India; 4 Division of Livestock Products Technology, Indian Veterinary Research Institute, Izatnagar, Bareilly, UP 243122 India

**Keywords:** DNA methylation, Epigenomics, Immune system, Milk proteins, Transcriptome

## Abstract

**Background:**

Subclinical mastitis (SCM) is a major constraint in dairy production and is driven by complex host–pathogen interactions. Although transcriptional responses associated with SCM have been widely investigated, the epigenetic mechanisms that stably regulate these programs remain less well characterized, particularly in crossbred cattle populations. This study aimed to characterize DNA methylation-based regulatory networks by integrating whole-genome methylation and transcriptome data from milk somatic cells of Vrindavani (*Bos taurus* × *Bos indicus)* cattle. Whole-genome methylation (*n* = 6) and corresponding transcriptome profiling (*n* = 6) were performed on milk somatic cells from SCM-affected and healthy control cows.

**Results:**

Differential methylation analysis (*q-*value < 0.05) identified 62,940 differentially methylated cytosines (DMCs), 7,706 differentially methylated regions (DMRs), and 6,203 differentially methylated genes (DMGs), with a predominant bias toward hypomethylation in SCM. Integrative analysis using stringent thresholds for both methylation (≥ 10%) and expression change (|log₂ fold change| ≥ 1; *P* of GMM < 0.001) identified 1,407 differentially methylated and expressed genes (DMEGs). Functional enrichment analysis revealed 47 KEGG pathways and 30 Gene Ontology biological process terms (FDR < 0.05), primarily associated with immune signaling and inflammatory responses. In contrast, a subset of DMEGs showed methylation-associated repression of lactation- and metabolism-related genes. Selected genes were experimentally validated by qPCR, including upregulation of the inflammatory mediator *S100A8* and downregulation of *CSN3* (κ-casein), a key milk protein gene.

**Conclusions:**

These findings provide an integrated view of the DNA methylation and transcriptional landscape of SCM in milk somatic cells and demonstrate that epigenetic remodeling is associated with coordinated activation of immune pathways alongside repression of lactation-associated functions. The results contribute to understanding the molecular basis of subclinical mastitis and may inform future efforts toward biomarker development and epigenetically informed strategies for improving disease resilience in dairy cattle.

**Graphical Abstract:**

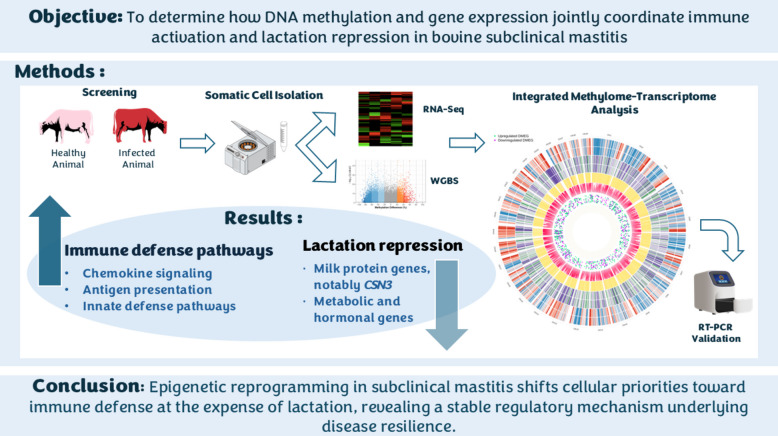

**Supplementary Information:**

The online version contains supplementary material available at 10.1186/s40104-026-01400-3.

## Background

Bovine mastitis continues to pose significant challenges for the global dairy sector, representing a major source of economic loss due to decreased production efficiency, increased veterinary expenses, and the early removal of affected animals from herds [1, 2]. The widespread use of antibiotics to manage the disease has also contributed to the growing threat of antimicrobial resistance, posing critical risks to both animal and public health [1–3]. Among its forms, subclinical mastitis (SCM) is particularly insidious; its lack of visible symptoms allows infections to persist chronically, silently compromising milk quality, decreasing yield, and impairing animal welfare [4]. Despite advances in herd management, the persistence of SCM highlights significant gaps in our understanding of mammary gland immunity.

The pathogenesis of mastitis involves a complex interplay between the invading pathogen and the host's innate and adaptive immune responses [5]. While modern genomic tools like genome-wide association studies (GWAS) and transcriptomics (RNA-seq) have identified key genes and pathways involved in the immune response, such as cytokine and Toll-like receptor signaling, they provide an incomplete picture [6, 7]. Traditional genetic approaches only partially explain an animal's susceptibility to mastitis, and transcriptomics, while providing a dynamic snapshot of gene expression, often fail to capture the stable regulatory mechanisms that underpin the chronic inflammatory state of SCM [8].

Epigenetics, particularly DNA methylation, is emerging as a crucial regulatory layer that can fill this knowledge gap. As a stable yet dynamic form of "cellular memory", DNA methylation modulates gene expression in response to external stimuli like pathogens without altering the underlying DNA sequence [9–11]. Recent studies have demonstrated that different mastitis-causing pathogens induce distinct DNA methylation signatures, effectively reprogramming the host's immune response [12, 13]. DNA methylation also has translational relevance. Unlike mRNA expression profiles, which are dynamic and vary with infection stage, methylation signatures are relatively stable and may serve as indicators of cumulative stress exposure or disease susceptibility [10, 14, 15]. Integrating methylation data with transcriptomic profiles may therefore support not only mechanistic understanding but also the development of diagnostic tools and breeding strategies for mastitis control. In parallel with immune activation, subclinical mastitis is consistently associated with compromised milk quality and reduced yield, reflecting altered regulation of lactation-associated pathways [4, 6]. Casein proteins are central determinants of milk composition, processing characteristics, and economic value, and reduced expression of casein genes has been reported during mastitis [16, 17]. However, the epigenetic mechanisms linking infection-associated immune activation to repression of milk protein synthesis remain poorly defined. Determining whether immune-driven epigenetic remodeling extends beyond defense pathways to core lactation genes may therefore provide a mechanistic link between molecular regulation and the phenotypic consequences of subclinical mastitis.

While this approach is gaining traction, the epigenetic landscape of mastitis in diverse cattle populations, particularly in economically important crossbreds, remains largely unexplored. Given that host responses can vary significantly by breed and geographical region, investigating naturally occurring mastitis in local herds is critical. Accordingly, the present study was designed to characterize the integrated DNA methylome and transcriptome of milk somatic cells from Vrindavani cattle, an important Indian crossbred (*Bos taurus* × *Bos indicus),* during naturally occurring subclinical mastitis. We aimed to investigate epigenetic regulatory networks that shape host immune responses and concurrently influence lactation-associated gene regulation, identify reproducible molecular signatures of disease, and provide a mechanistic foundation for the development of biomarkers and improved management strategies.

## Methods

### Experimental animals, phenotypic stratification, and sample procurement

All experimental procedures involving animals were reviewed and approved by the Institutional Animal Ethics Committee (IAEC). The study cohort comprised multiparous Vrindavani crossbred dairy cows from a single herd. All animals were housed and managed under the farm’s standard nutritional and husbandry protocols, thereby minimising non-experimental variation and allowing reliable cohort comparisons.

To establish distinct healthy and SCM cohorts, lactating cows (*n* = 118) were subjected to a rigorous, longitudinal screening process over three consecutive months. Initial screening was performed using the California Mastitis Test (CMT) [18]. Subsequently, definitive quantification of milk somatic cell count (SCC) was conducted using the Direct Microscopic Somatic Count (DMSCC) method [19]. Animals were stratified into two experimental groups based on the consistent exhibition of specific SCC thresholds: the SCM group (SCC ≥ 200,000 cells/mL) and the Healthy Control (HC) group (SCC ≤ 150,000 cells/mL) [20]. Although the diagnostic cutoff for subclinical mastitis was defined as an SCC ≥ 200,000 cells/mL, the animals selected for the SCM group consistently exhibited SCC values in the range of 4–5 × 10^5^ cells/mL across repeated monthly measurements, whereas healthy control animals remained below 150,000 cells/mL throughout the screening period. Further stringency was applied through specific inclusion criteria: only multiparous animals (parity ≥ 2) in their mid- to late-lactation phase (≥ 92 days in milk) were selected. Exclusion criteria included any documented clinical disease within the preceding year or any therapeutic intervention within the six months before sample collection [21, 22]. Milk samples were collected aseptically before morning milking. Following the teat disinfection and discarding the initial milk streams, samples for downstream analysis were obtained [23]. For the SCM group, 200 mL of milk was collected from a single representative quarter reflecting the localized nature of subclinical mastitis. For the HC group, a 200 mL composite sample representing the baseline healthy mammary state was prepared by pooling equal volumes (50 mL) from all four quarters. Samples were immediately placed on ice for transport to the laboratory.

Upon arrival, milk somatic cells were isolated, and an initial low-speed centrifugation at 1,500 × *g* for 15 min at 4 °C was performed to separate the layers. After discarding the upper fat and whey, the resulting cell pellet was purified with two sequential washes, each involving the addition of 40 mL of phosphate-buffered saline followed by centrifugation under the same conditions. The purified milk somatic cell pellet was subsequently divided into two aliquots for nucleic acid preservation: one fraction was stored at −20 °C for genomic DNA extraction, while the second was stabilized in TRIzol™ reagent and stored at −80 °C to maintain RNA integrity for subsequent transcriptomic analysis [24].

### DNA isolation and methylome data sequencing from milk somatic cells

Genomic DNA was isolated from the Somatic cells (*n* = 6) using the DNeasy Blood and Tissue Kit (Qiagen Inc., Toronto, ON, Canada). Subsequently, whole-genome methylation sequencing (WGMS) libraries were prepared using the NEBNext^®^ Enzymatic Methyl-seq kit (New England BioLabs Ltd., Whitby, ON, Canada), an enzymatic method selected for its high accuracy [23]. Qubit and the QIAxcel Advanced system assessed library quality, and libraries meeting quality criteria were further validated with TapeStation Analysis Software 5.1. The sequencing was performed on an Illumina HiSeq 2000 platform, generating paired-end reads at an average depth of 20X to ensure comprehensive genome-wide methylation profiling.

### Bioinformatic analysis of whole-genome bisulfite sequencing data

Raw whole-genome bisulfite sequencing (WGBS) data were processed using the open-source nf-core/methylseq pipeline tools [25]. Initial quality control was conducted with FastQC (v0.11.9), after which adapter sequences and low-quality bases (Phred score < 30) were systematically removed using Trim Galore! (v0.6.6). To mitigate potential bias introduced by enzymatic end-repair, 8 bp were trimmed from both ends of each read.

The resulting high-quality, clean reads were aligned to the *Bos taurus* reference genome (ARS-UCD2.0) using Bismark (v0.22.0), which implements the Bowtie2 aligner optimized for bisulfite-converted DNA. Single-base resolution methylation profiles were then generated using the Bismark methylation extractor module [26]. Only cytosine positions with a minimum sequencing depth of ≥ 7 × in every individual sample were retained before merging CpG sites across samples for downstream analysis. Applying this minimum coverage threshold helps ensure reliable methylation estimates while retaining sufficient CpG sites for robust genome-wide differential methylation analysis.

Differential methylation analysis was performed using the MethylKit package (v3.12) in R [27]. To control for potential confounding effects, parity and lactation stage were incorporated as covariates in the statistical model. Differentially methylated cytosines (DMCs) were identified based on two criteria: an absolute methylation difference of ≥ 20% between experimental groups and a *q*-value < 0.05*.* Subsequently, differentially methylated regions (DMRs) were identified using a 1-kb sliding window approach. A region was designated a DMR if it exhibited a mean methylation difference of ≥ 20%, contained a minimum of three significant DMCs, and had a *q*-value < 0.05.

### RNA isolation, sequencing, and raw data processing

Total RNA was extracted from milk somatic cells (*n* = 6) using TRIzol™ reagent (Thermo Fisher Scientific, Waltham, MA, USA). RNA quality and integrity were assessed using the Agilent 2100 Bioanalyzer (Agilent Technologies, Santa Clara, CA, USA), and only QC-passed samples were then used for subsequent library preparation and sequencing. Subsequently, sequencing libraries were constructed from high-quality RNA samples with the NEBNext® Ultra™ II RNA Library Prep Kit (New England BioLabs, Ipswich, MA, USA). The final libraries were quantified via Qubit (Thermo Fisher Scientific, Waltham, MA, USA) and validated with a TapeStation system (Agilent Technologies, Santa Clara, CA, USA) before undergoing paired-end sequencing on an Illumina platform (Illumina, Inc., San Diego, CA, USA) for subsequent transcriptomic analysis.

Initial quality control of the raw paired-end sequencing reads was performed using FastQC (v 0.12.1). Subsequently, the fastp toolkit (v 0.23.4) was employed for adapter detection, trimming, and quality filtering. All fastp parameters were left at default settings, except for the quality-filtering threshold, which was set to remove reads with a Phred score below 30 [28]. The resulting high-quality, clean reads were then aligned to the *Bos taurus* reference genome (ARS-UCD2.0) using the splice-aware STAR aligner (v 2.7.11b) with its default parameters [29]. Following alignment, transcript-level abundance was quantified using Salmon (v1.10.2) in its alignment-based mode. Gene-level expression values were subsequently obtained by summarizing transcript abundances and expressed as transcripts per million (TPM). These TPM values represent normalized expression estimates that account for transcript length and sequencing depth and were used as input for downstream methylation–transcriptome integration analyses using the MethGET framework [30, 31].

### Methylation and transcriptome data integration and identification of differentially methylated and expressed genes (DMEGs)

To elucidate the regulatory interplay between DNA methylation and transcription, we performed a multi-layered integrative analysis using the MethGET program [31]. The workflow integrated three primary data types: single-base resolution Differential methylation profiles in CGmap format, Expression matrices, and the *Bos taurus* gene annotation file (RefSeq assembly GCF_002263795.3). The analytical pipeline began with annotating essential genomic features, including gene bodies, exons, introns, and promoters, with promoters defined as the 1-kb region immediately upstream and the 500 bp region downstream of the transcription start site (TSS). The left and right CGI shores were designated as the 2,000 bp regions directly upstream and downstream of CpG islands (CGIs). Similarly, the left and right CGI shelves were defined as the 2,000 to 4,000 bp regions upstream and downstream of each corresponding CGI. For each gene, the average methylation level across these distinct features was determined by aggregating the methylation values of all constituent CpG sites.

The core of the integrative analysis was conducted using the MethGET program to identify genes under direct epigenetic regulation [31]. An initial exploratory analysis was conducted by calculating sample-wise Pearson correlations to evaluate the baseline relationship between regional methylation and gene expression. To investigate coordinated changes between the experimental groups, we first performed a genome-wide correlation analysis between differential methylation and expression. Subsequently, to robustly identify genes with significant concerted changes, we employed a Gaussian Mixture Model (GMM) implemented in Python’s scikit-learn library [32]. In this framework, the GMM is used as an unsupervised probabilistic model to group genes based on joint methylation and expression patterns aggregated across regulatory regions. Model output is expressed as posterior probabilities reflecting the strength of assignment to the inferred mixture components. In this study, this posterior probability is reported as “*P *of GMM”, representing the probability that a gene belongs to the regulatory component characterized by coordinated methylation–expression changes.

Genes were filtered and classified as differentially methylated and expressed genes (DMEGs) only if they simultaneously satisfied stringent statistical thresholds for both expression (|log₂FC| ≥ 1) and methylation (≥ 10% change). In addition, genes were required to meet a stringent GMM assignment threshold (*P *of GMM < 0.001). Here*, P *of GMM denotes the posterior probability from the Gaussian mixture model, reflecting the confidence in assigning the gene to the regulatory component characterised by coordinated methylation–expression changes. This threshold was used to retain only high-confidence genes exhibiting concordant epigenetic and transcriptional regulations. This classification was contingent upon the methylation change occurring within a key regulatory region, specifically, the promoter, first exon, or first intron. We conducted separate GMM analyses for each of these three regulatory regions to dissect the precise locus of epigenetic control.

### Functional enrichment and pathway analysis

To clarify the biological relevance of the observed molecular alterations, we carried out functional enrichment analysis for each gene set. Analyses were performed in R using the clusterProfiler package (v4.2.2) [33] and independently verified with DAVID Bioinformatics Resources (v6.8) [34] and g:Profiler [35]. Gene Ontology (GO) terms and Kyoto Encyclopedia of Genes and Genomes (KEGG) pathways were systematically evaluated, and only those meeting a stringent FDR-adjusted *P-*value threshold (< 0.05) were considered significant. Enrichment results were visualized using ggplot2 and GOplot [35], while global patterns and pathway-level relationships were depicted through OmicCircos [36] for integrative circular genome plots and ComplexHeatmap [37] for clustered heatmap visualizations.

### Validation of promoter-associated differentially methylated genes by quantitative real-time PCR

To validate promoter-associated epigenetic regulation, immune-related DMEGs were selected for experimental confirmation by quantitative real-time PCR (qRT-PCR). Total RNA was isolated from milk somatic cells using TRIzol reagent (Invitrogen) according to the guanidinium thiocyanate–phenol–chloroform method, and cDNA synthesis was performed with the QuantiTect Reverse Transcription Kit (Qiagen), which includes an integrated genomic DNA removal step. Gene-specific primers were designed with the PrimerQuest™ tool (Integrated DNA Technologies) and evaluated using OligoAnalyzer to ensure optimal parameters. Lyophilized primers were reconstituted to 100 µmol/L stock solutions and diluted to 10 µmol/L working solutions for storage at −20 °C. Annealing conditions were optimized using gradient PCR across 50–60 °C to confirm specificity. qRT-PCR was performed in compliance with Minimum Information for Publication of Quantitative Real-Time PCR Experiments (MIQE) guidelines [38], using SYBR Green detection chemistry in 10 µL reactions containing 5 µL of 2 × SYBR Green Master Mix, 0.5 µL of each primer (10 µmol/L), 0.5 µL of cDNA (~ 15 ng), and nuclease-free water. The cycling protocol consisted of an initial denaturation at 95 °C for 3 min, followed by 40 cycles of 95 °C for 5 s and 60 °C for 30 s, with melt-curve analysis (65–95 °C) to verify amplification specificity. All assays were run in triplicate for three biological replicates per group, with *GAPDH* used as the internal reference gene. Expression levels were quantified using the 2^−∆∆Ct^ method [39], and results were expressed as log₂ fold changes.

## Results

### Epigenome-wide DNA hypomethylation and functional consequences in subclinical mastitis

#### Global DNA hypomethylation characterizes the methylome of subclinical mastitis

**Fig. 1:**
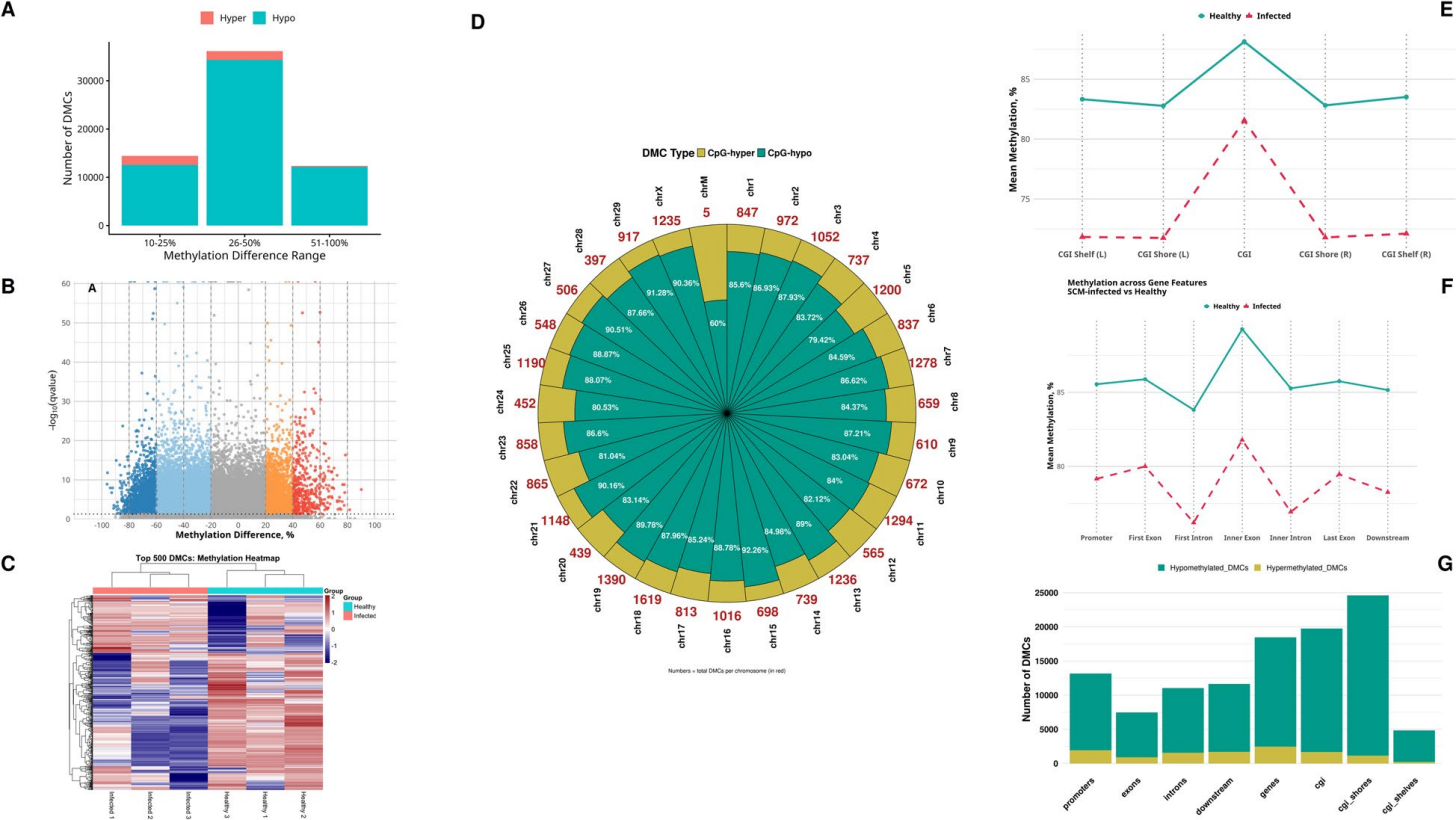
Epigenome-wide DNA hypomethylation in subclinical mastitis (SCM). **A** Distribution of DMCs by magnitude of methylation difference, highlighting the predominance of large hypomethylation events. **B** Volcano plot of DMCs displaying (methylation difference, %) versus statistical significance (−log₁₀ *q*-value), with hypomethylated cytosines predominating. **C** Heatmap of the top 500 DMCs, separating HC and SCM samples by methylation profile. **D** Chromosomal distribution of DMCs, showing density and the proportion of hypomethylation (inner ring) vs. hypermethylation (outer ring) for each chromosome, revealing preferential targeting of CpG island (CGI) shores and shelves. **E**–**F** Mean methylation levels across CGI features and gene structures in healthy control (HC) vs. SCM cows, showing marked reductions in SCM, especially in first introns and downstream regions. **G** Genomic feature distribution of hypomethylated vs. hypermethylated DMCs, revealing preferential targeting of CGI shores and shelves

To investigate the epigenetic landscape of SCM, we performed whole-genome methylation sequencing on milk somatic cells from three SCM-positive (SCP) and three healthy control (HC) cows. The analysis yielded high-quality data, with an average of 85.1% of reads aligning to the bovine reference genome (Table S1, Fig. S1). A global assessment revealed that milk somatic cells in the HC group maintained high methylation levels across all genic features (85.0%–89.0%). In comparison, SCM-infected samples exhibited a significant and consistent reduction in DNA methylation, with average levels dropping to 74.3%–81.5% (Table S2). This loss of methylation was observed across promoters, gene bodies, and CGI structures, with the most pronounced decreases occurring in the first intron and downstream regions (Fig. 1E and F).

#### Identification of differentially methylated cytosines (DMCs) and regions (DMRs)

We identified 62,940 DMCs between the SCP and HC groups (Table S3). A prominent feature of these DMCs was the overwhelming predominance of hypomethylation in the SCM samples. Of the total DMCs, 59,045 (93.8%) were hypomethylated, while only 3,895 (6.2%) were hypermethylated (Fig. 1B and C). This pattern was consistent at a regional level, where we identified 7,706 DMRs (Fig. S2, Table S4). Among these, 7,617 (98.8%) were hypomethylated in the SCM group, reinforcing the observation of a genome-wide erosion of methylation associated with the disease state. When stratified by the magnitude of methylation change, most sites had a substantial methylation difference of 25%–50% and were almost exclusively hypomethylated (98.5%), suggesting that large-scale epigenetic shifts in SCM are overwhelmingly demethylation events (Fig. 1A).

#### Chromosomal landscape reveals DMC hotspots and a pervasive hypomethylation bias

While DMCs were distributed across the entire bovine karyotype, their density was non-uniform, revealing distinct chromosomal hotspots (Fig. 1D). Chromosome 18 harbored the highest number of DMCs (*n* = 1,619), followed by Chr19 (*n* = 1,390) and Chr11 (*n* = 1,294). Several other autosomes and the X chromosome also displayed a high density of DMCs (> 1,200).

A pervasive hypomethylation bias was observed across all chromosomes. The proportion of hypomethylated DMCs on individual chromosomes ranged from a low of 79.4% on Chr5 to a high of 92.3% on Chr15 and Chr29. Despite this overwhelming trend, we also noted localized exceptions. Chromosomes 22 and 24, for instance, exhibited a relatively higher proportion of hypermethylation (~ 19% each). This finding suggests that while global demethylation is the primary epigenetic signature of SCM, more complex, locus-specific hypermethylation events also occur on chromosomes.

#### Preferential demethylation occurs at key regulatory and genic locations

An analysis of the genomic location of DMCs revealed a clear pattern of preferential targeting (Fig. 1G). The highest concentration of DMCs was found in CGI shores (24,564 DMCs), followed by gene bodies (18,455 DMCs) and promoters (13,147 DMCs). The ratio of hypomethylated to hypermethylated DMCs was most extreme in CGI shores (~ 20.5-fold enrichment) and CGI shelves (~ 20.9-fold). This intense, targeted demethylation at the flanking regions of CpG islands, which are known to be critical for gene regulation, is consistent with the findings of previous similar studies and strongly indicates a directed epigenetic mechanism aimed at altering the expression of nearby genes in response to SCM.

#### Functional annotation of hypomethylated and hypermethylated Genes

A total of 6,203 unique genes carrying differentially methylated genes (DMGs) were identified (FDR ≤ 0.05) (Table S5). Among these, 807 genes contained more than five DMCs, 243 genes carried more than ten, and 109 genes had more than fifteen DMCs, indicating concentrated regions of methylation change.

**Fig. 2:**
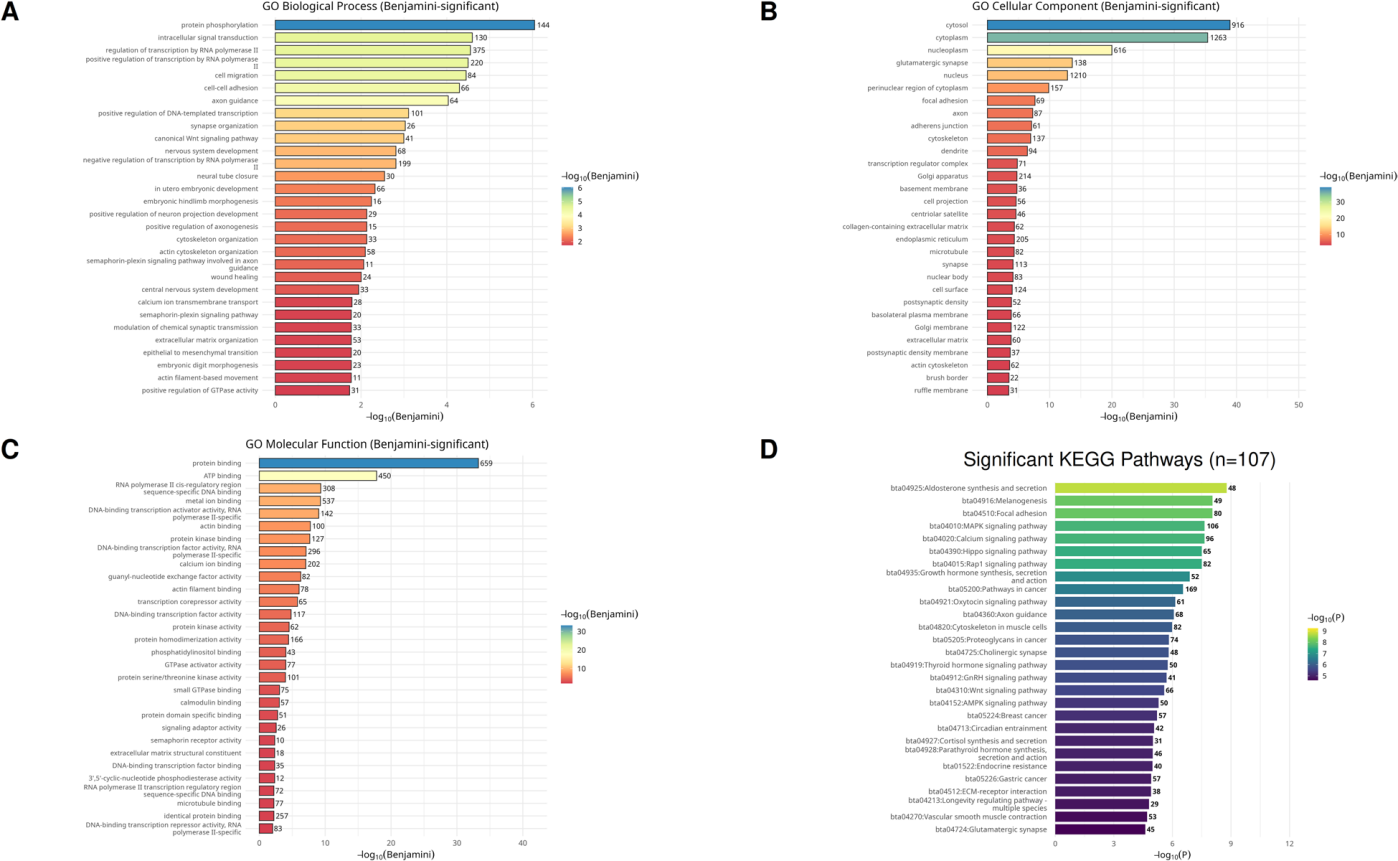
Functional annotation of hypomethylated genes in subclinical mastitis. **A** GO biological process enrichment highlights immune signaling cascades (B and T cell receptor pathways) alongside developmental processes such as neurogenesis and axon guidance. **B** GO Cellular Component terms reveal enrichment of cytosolic, nuclear, and mitochondrial compartments, as well as adhesion complexes, cytoskeletal elements, and synaptic structures. **C** GO molecular function categories are dominated by protein, ATP, ion, actin, and DNA binding, together with transcriptional regulators and diverse kinase activities. **D** KEGG pathway analysis demonstrates significant enrichment of immune and intracellular signaling networks

Within the subset of 5,280 hypomethylated genes, functional annotation revealed significant enrichment in multiple biological processes and pathways. In total, 136 KEGG pathways and 173 GO terms were significantly enriched (FDR ≤ 0.05), comprising 117 Biological Process (BP) terms, 27 Molecular Function (MF) terms, and 29 Cellular Component (CC) terms (Table S6). The hypomethylated genes were associated with immune signaling as well as developmental processes. GO Biological Process terms included *B cell receptor signaling pathway* and *T cell receptor signaling pathway*, together with developmental categories such as *neurogenesis* (adjusted *P* = 2.8 × 10^−^^6^) and *axon guidance*. These observations indicate that genes linked to both immune regulation and developmental remodeling were affected (Fig. 2A).

The GO Cellular Component profile included enrichment in cytosol, nucleoplasm, mitochondria, Golgi apparatus, and endoplasmic reticulum, along with adhesion complexes, cytoskeletal structures, and synaptic components (Fig. 2B). GO Molecular Function terms were dominated by binding activities (protein, ATP, ion, actin, DNA) and transcriptional regulation, including RNA polymerase II-related activators and repressors. Several kinase activities were also represented, including protein, serine/threonine, and histone-specific kinases (Fig. 2C).

KEGG pathway analysis showed that hypomethylated genes were enriched in immune signaling cascades such as *B cell receptor* (*q* = 0.00174) and *T cell receptor* (*q* = 0.02264), along with pathways regulating intracellular signaling and endocrine functions, including *MAPK signaling*, *Calcium signaling*, *Wnt*, *Hippo*, *Rap1*, *AMPK*, *mTOR*, and *FoxO*. Neuroendocrine and synaptic pathways, as well as metabolic and longevity-associated processes, were also represented (Fig. 2D).

Among the 923 hypermethylated genes, no KEGG pathways or GO terms reached significance at the applied FDR threshold.

### Identification of differentially methylated and expressed genes (DMEGs)

**Fig. 3:**
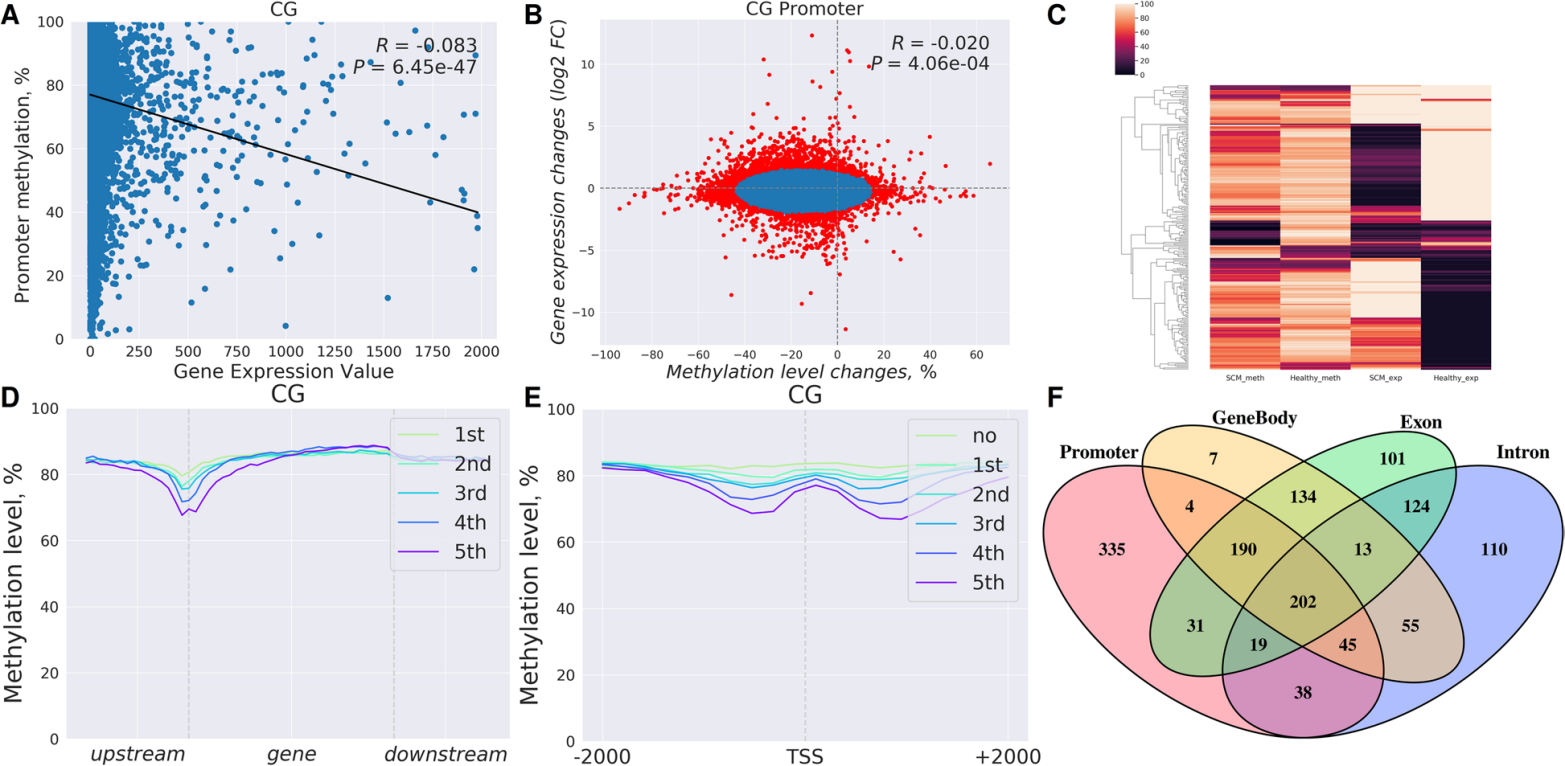
Identification and correlation analysis of differentially methylated and expressed genes (DMEGs). **A** Genome-wide negative correlation between promoter methylation percentage and gene expression values, underscoring the classical epigenetic relationship between DNA demethylation and transcriptional activation. **B** Scatterplot of promoter methylation differences versus gene expression fold changes, showing a weak but significant inverse correlation (*R* = –0.020, *P* = 4.06 × 10⁻^4^). **C** Heatmap of methylation and expression values for DMEGs, clearly separating healthy and SCM groups and illustrating the coordinated pattern of hypomethylation with upregulation and hypermethylation with downregulation. **D**–**E** Meta-gene and TSS plots of methylation levels across genic regions, illustrating reduced methylation in SCM samples across regulatory elements, particularly near transcription start sites. **F** Venn diagram showing overlap of DMEGs across promoters, exons, introns, and gene bodies. A substantial number of genes exhibit methylation changes in multiple regions, with 148 affected simultaneously in all three major regulatory contexts

To comprehensively assess the interplay between DNA methylation and transcriptional regulation, by applying a GMM, we identified 1,922 genes exhibiting significant changes in promoter DNA methylation and gene expression (*P *of GMM < 0.001*)*. Of these, 857 genes displayed both substantial promoter methylation alterations (> 10%) and marked transcriptional changes (|log₂ fold change| > 1) and were classified as DMEGs. Using parallel criteria, we identified 813 and 605 DMEGs for exons and introns, respectively. By merging these sets, a total of 1,407 unique DMEGs were detected, including 148 genes exhibiting significant methylation changes simultaneously across promoters, exons, and introns (Fig. 3B, F, and Table S7).

Among these DMEGs, approximately 842 displayed inverse relationships between DNA methylation and gene expression, consistent with classical epigenetic regulatory paradigms. Specifically, 88 DMEGs demonstrated hypermethylation across regulatory regions concomitant with downregulation of expression.

### Correlation analyses between methylation and gene expression changes

To further investigate these relationships, we examined correlations between DNA methylation changes and gene expression alterations across different genic features. Methylation changes within exons showed a weak but highly significant inverse correlation with changes in gene expression (*R* = −0.043, *P* = 3.40 × 10^–13^). Similar patterns were observed for methylation changes in gene bodies (*R* = −0.051, *P* = 2.67 × 10^–18^), introns (*R* = −0.036, *P* = 1.73 × 10^–8^), and promoters (*R* = −0.020, *P* = 4.06 × 10^–4^). Together, these analyses indicate consistent genome-wide trends linking methylation variation to transcriptional changes, despite modest correlation coefficients (Fig. 3B, D, and F).

### Integrated epigenomic and transcriptomic analysis reveals a coordinated immune response in subclinical mastitis

#### Functional annotation of upregulated differentially methylated and expressed genes

**Fig. 4:**
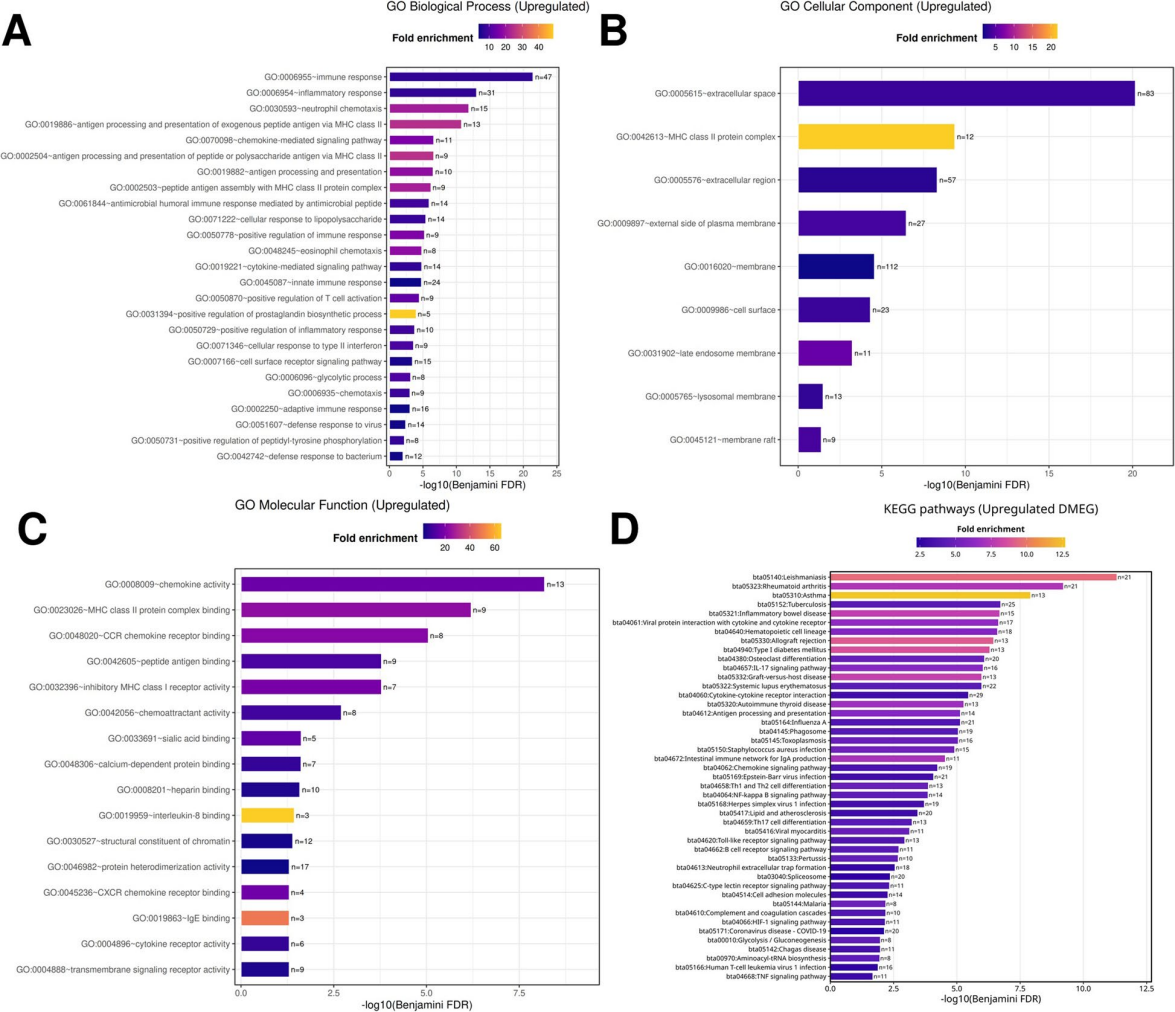
Functional enrichment of upregulated differentially methylated and expressed genes (DMEGs) highlights coordinated immune activation in subclinical mastitis. **A** GO Biological Process terms show strong representation of immune and inflammatory responses, innate and adaptive immune processes, neutrophil chemotaxis, and responses to lipopolysaccharide. **B** GO Cellular Component analysis reveals enrichment of extracellular space, MHC class II protein complex, plasma membrane, and lysosomal compartments. **C** GO Molecular Function categories include protein heterodimerization activity, chemokine activity, peptide antigen binding, MHC class II binding, and diverse cytokine and receptor interactions. **D** KEGG pathway analysis identifies enrichment of infection- and immunity-related pathways, including Leishmaniasis, Rheumatoid arthritis, Asthma, and antigen presentation and cytokine signaling cascades

Functional enrichment analysis of the 801 upregulated DMEGs identified 47 KEGG pathways (FDR ≤ 0.05). The most enriched pathways included *Leishmaniasis*, *Rheumatoid arthritis*, *Asthma*, and other immune- and infection-related processes. The enrichment profile comprised infectious disease pathways, autoimmune and inflammatory signaling cascades, antigen presentation, cytokine and chemokine signaling, and lymphocyte differentiation (Fig. 4D).

Analysis of GO Biological Processes yielded 30 significant terms (FDR ≤ 0.05). The most represented categories were *immune response* (*n* = 47), *inflammatory response* (*n* = 31), and *innate immune response* (*n* = 24). Additional terms included *adaptive immune response*, *neutrophil chemotaxis*, *cell surface receptor signaling*, and *cellular response to lipopolysaccharide*. Antigen presentation-associated categories such as *MHC class II peptide antigen presentation* and *antimicrobial humoral immune response* were also identified (Fig. 4A).

GO Cellular Component analysis identified nine enriched terms, the most prominent being *extracellular space* (*n* = 83) and *MHC class II protein complex* (*n* = 12, ~ 20-fold enrichment). Other significant categories included *extracellular region* (*n* = 57), *external side of plasma membrane* (*n* = 27), *cell surface* (*n* = 23), *lysosomal membrane* (*n* = 13), and *membrane raft* (*n* = 9) (Fig. 4B).

For GO Molecular Function, 16 enriched terms were observed. The most represented were *protein heterodimerization activity* (*n* = 17) and *chemokine activity* (*n* = 13). Other enriched terms included *structural constituent of chromatin* (*n* = 12), *peptide antigen binding* (*n* = 9), *MHC class II protein complex binding* (*n* = 9), *transmembrane signaling receptor activity* (*n* = 9), and multiple receptor–ligand interactions such as *chemokine receptor binding* (*n* = 8), *cytokine receptor activity* (*n* = 6), and *interleukin-8 binding* (*n* = 3) (Fig. 4C).

**Fig. 5:**
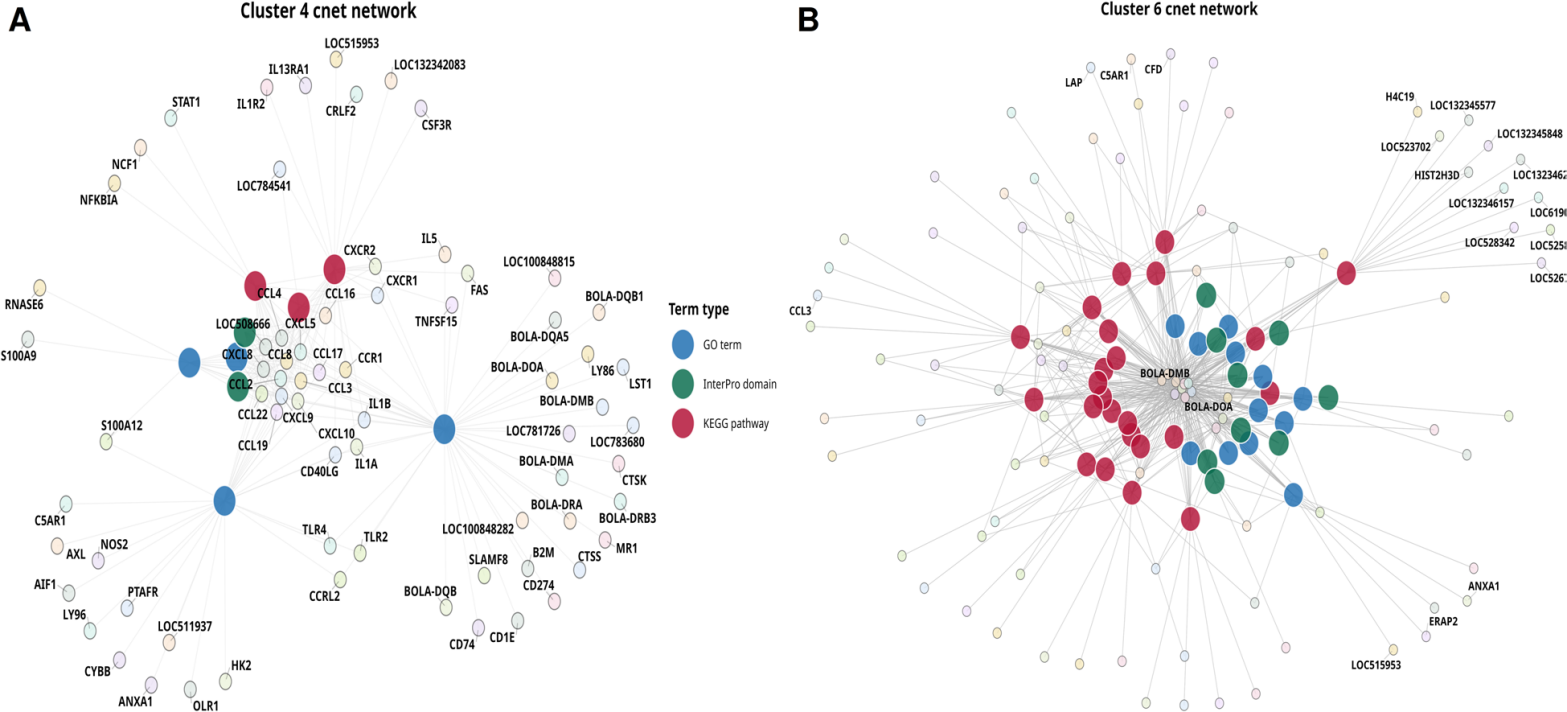
Functional annotation clustering of upregulated differentially methylated and expressed genes (DMEGs) reveals coordinated immune activation. **A** Cluster 4 network showing strong enrichment for immune and inflammatory responses, chemokine activity, and cytokine–cytokine receptor interaction. **B** Cluster 6 network enriched for antigen processing and presentation via MHC class II, with links to infection-related pathways such as tuberculosis, influenza A, and *Staphylococcus aureus* infection

Cluster analysis revealed 30 significant annotation clusters (Enrichment Score > 1.3, FDR ≤ 0.05) (Table S8). The highest-scoring cluster (ES = 16.2) was enriched for *extracellular space*, *extracellular region*, and *secreted proteins*. Cluster 2 (ES = 11.5) highlighted *chemotaxis* and *neutrophil chemotaxis*, while Cluster 4 (Fig. 5A) (ES = 10.3) included *immune response*, *inflammatory response*, *chemokine activity*, and *cytokine–cytokine receptor interaction*, involving genes such as *CXCL8*, *CXCL9*, *CXCL10*, *CCL2*, *CCL3*, *CCL4*, and *CCL5* with their receptors *CCR1*, *CCR2*, *CCR5*, and *CXCR2*. Cluster 6 (ES = 6.8) showed enrichment for *antigen processing and presentation *via* MHC class II*, including *CD74*, *BOLA-DRA*, *BOLA-DRB3*, *CTSB*, and *CTSS*, with overlap into infection-related pathways such as *tuberculosis*, *influenza A*, and *Staphylococcus aureus infection* (Fig. 5B).

Other clusters included Cluster 7 (*immunoglobulin-like domains* and *B cell receptor signaling*), Cluster 9 (*chemokine receptor binding*), Cluster 10 (*eosinophil chemotaxis*), Cluster 15 (*antimicrobial responses*), Cluster 14 (*glycolysis/gluconeogenesis*), and Clusters 23/28 (*Toll-like receptor signaling*, *NETosis*, and chromatin structural proteins).

Together, these results indicate that the upregulated DMEGs were associated with immune signaling pathways, chemotaxis, antigen processing and presentation, extracellular communication, and metabolic remodeling.

#### Functional annotation of downregulated differentially methylated and expressed genes

The 606 downregulated DMEGs showed limited functional enrichment. No KEGG pathways met the FDR threshold (≤ 0.05). GO analysis identified three significant Biological Process (BP) terms: *G protein–coupled receptor signaling pathway* (*n* = 49), *response to dehydroepiandrosterone* (*n* = 4), and *response to 11-deoxycorticosterone* (*n* = 4).

GO Cellular Component (CC) analysis yielded three enriched terms: *extracellular region (n* = 42), *Golgi lumen* (*n* = 4), and *extracellular space* (*n* = 42). GO Molecular Function (MF) analysis identified two categories: *G protein–coupled receptor activity* (*n* = 39) and *olfactory receptor activation* (*n* = 39).

**Fig. 6:**
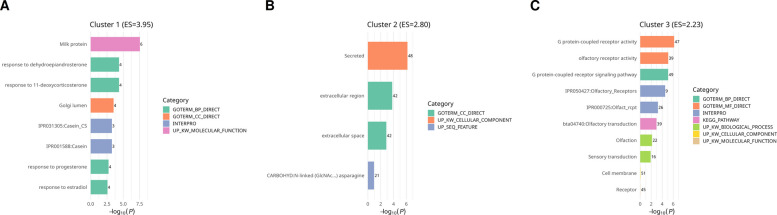
Functional annotation clustering of downregulated differentially methylated and expressed genes (DMEGs) reveals hormone responsiveness, extracellular localization, and GPCR signaling. **A** Cluster 1 (Enrichment Score = 3.95) enriched for milk protein genes and hormone-responsive processes, including responses to dehydroepiandrosterone, 11-deoxycorticosterone, progesterone, and estradiol, together with Golgi lumen localization and casein-related protein domains. **B** Cluster 2 (Enrichment Score = 2.80) enriched for secreted proteins, extracellular region, extracellular space, and N-linked glycosylation features. **C** Cluster 3 (Enrichment Score = 2.23) enriched for G protein–coupled receptor (GPCR) activity, olfactory receptor activation, and GPCR signaling pathways, with KEGG annotation mapping to olfactory transduction

Cluster analysis of the downregulated gene set revealed three annotation clusters (Enrichment Score > 1.3, FDR ≤ 0.05). Cluster 1 (Enrichment Score = 3.95) was enriched for *milk protein genes* and *hormone-responsive processes*, including *responses to dehydroepiandrosterone, 11-deoxycorticosterone, progesterone,* and *estradiol*, alongside *Golgi lumen localization* and *casein-related protein domains*. Cluster 3 (Enrichment Score = 2.23) included *G protein–coupled receptor activity*, *olfactory receptor activity*, and the *GPCR signaling pathway*, with KEGG annotation mapping to *olfactory transduction*. Cluster 2 (Enrichment Score = 2.80) was associated with *secreted proteins*, *extracellular region*, *extracellular space*, and *N-linked glycosylation motifs* (Fig. 6A–C).

Overall, the downregulated DMEGs were associated with gene sets related to hormone/steroid responses, GPCR-mediated signalling, extracellular localisation, and milk protein functions, consistent with suppression of lactation-associated pathways during subclinical mastitis.

**Fig. 7:**
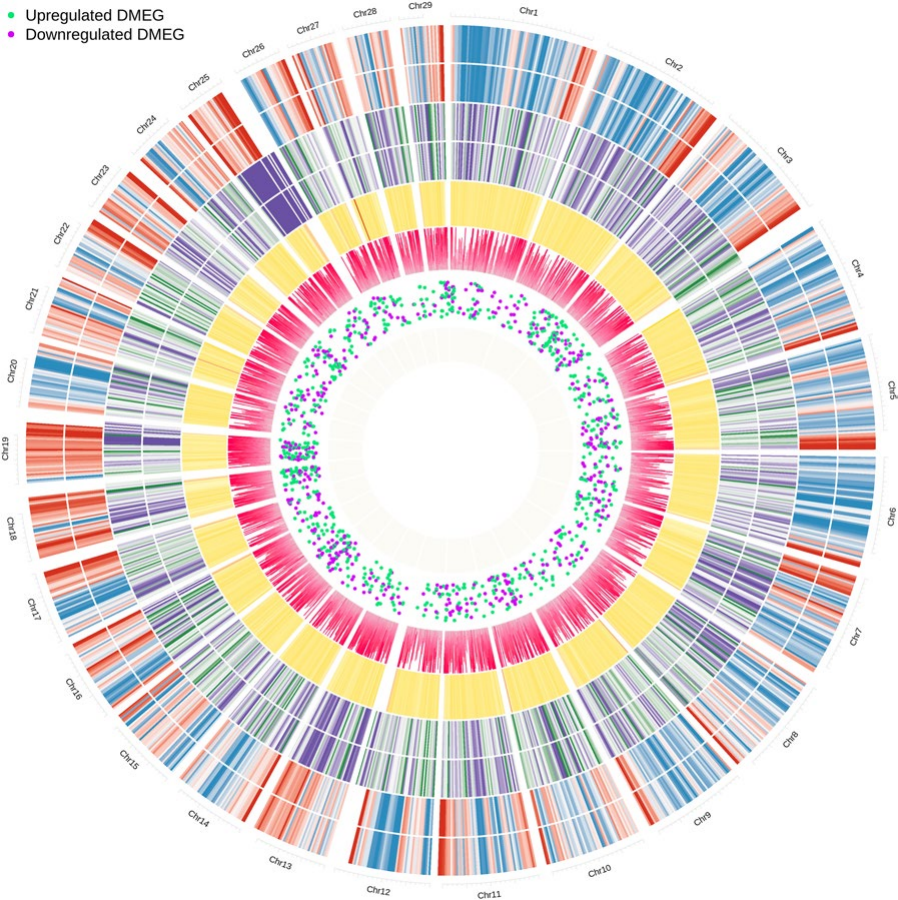
Integrated epigenomic and transcriptomic landscape of subclinical mastitis. The Circos plot provides a genome-wide overview of molecular changes across the bovine autosomes (outermost ring). The concentric tracks, from outside to inside, display heatmaps of absolute DNA methylation and gene expression in healthy control and SCM groups, followed by a heatmap of differential methylation (Δ-methylation) highlighting hypomethylated versus hypermethylated regions. The inner tracks represent a Manhattan plot of the statistical significance of differentially methylated cytosines (DMCs) and a scatter plot showing the genomic locations of upregulated (green) and downregulated (purple) differentially methylated and expressed genes (DMEGs)

An integrated, genome-wide visualization of the data revealed a profound and widespread reprogramming of the epigenome and transcriptome in response to SCM. These molecular alterations were distributed across all autosomes but concentrated in distinct chromosomal hotspots, with a clear bias towards DNA hypomethylation and clusters of highly significant differential methylation (Fig. 7).

### qPCR analysis verifies gene regulation mediated by promoter methylation

**Fig. 8:**
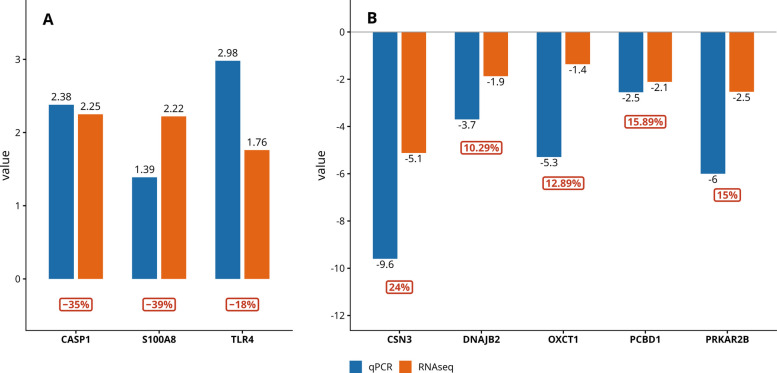
qPCR validation confirms promoter methylation-mediated regulation of gene expression in subclinical mastitis. **A** Upregulated genes (*S100A8, TLR4, CASP1*) show concordant increases in expression between RNA-Seq and qPCR, consistent with promoter hypomethylation. **B** Downregulated genes (*OXCT1, PCBD1, DNAJB2, PRKAR2B, CSN3*) exhibit strong agreement between RNA-Seq and qPCR, in line with promoter hypermethylation

To evaluate whether epigenetic remodeling associated with immune activation also extends to repression of lactation-related genes, we selected representative differentially methylated and expressed genes for experimental validation. RT-qPCR confirmed the direction and magnitude of expression changes observed in RNA-Seq across all eight selected genes, with promoter hypomethylation generally associated with transcriptional upregulation (*S100A8, TLR4, CASP1*) and promoter hypermethylation linked to downregulation (*CSN3, PRKAR2B, OXCT1, PCBD1, DNAJB*2). Notably, *CSN3* and *PRKAR2B* exhibited particularly robust concordance between methylation, RNA-Seq, and qPCR results (*P* = 0.011 and *P* = 0.003, respectively), further reinforcing their role as key epigenetically regulated genes during subclinical mastitis. Despite expected differences in effect size between sequencing and qPCR platforms, the overall patterns remained consistent, confirming that promoter methylation status reliably predicts transcriptional changes in this context (Fig. 8A and B, Table 1).

**Table 1:** qPCR validation of selected DMEGs and corresponding promoter methylation changes

**Gene**	**RNA-Seq log₂FC**	**qPCR log₂FC**	**% Promoter methylation change**
**Hypomethylated and upregulated genes**			
* S100A8*	+ 2.22	+ 1.39	− 39%
* TLR4*	+ 1.76	+ 2.98	− 18%
* CASP1*	+ 2.25	+ 2.38	− 35%
**Hypermethylated and downregulated genes**			
* CSN3*	− 5.12	− 9.6	+ 24%
* PRKAR2B*	− 2.53	− 6.0	+ 15%
* OXCT1*	− 1.36	− 5.3	+ 12.9%
* PCBD1*	− 2.11	− 2.55	+ 15.9%
* DNAJB2*	− 1.87	− 3.7	+ 10.3%

## Discussion

Subclinical mastitis represents one of the most persistent and economically damaging challenges in the dairy sector because of its hidden nature and chronic course [4]. Our integrative analysis shows that the pathology of SCM is defined not only by sustained immune activation but also by profound epigenetic remodeling across the bovine genome. We observed abundant DNA methylation changes and a pervasive bias toward hypomethylation, particularly at promoters and CpG island shores, which was strongly associated with the transcriptional activation of immune response genes. Conversely, focal promoter hypermethylation was linked to the repression of milk protein synthesis and metabolic genes, indicating a deliberate trade-off between immune defense and lactation-associated production functions. To the best of our knowledge, this is the first study in crossbred cattle to investigate the integration of genome-wide DNA methylation with transcriptome data from milk somatic cells. The results indicate that SCM encompasses coordinated epigenetic reprogramming, rather than mere pathogen invasion, thereby providing valuable insights into the fundamental epigenetic mechanisms underlying transcriptional alterations. This supports the hypothesis that DNA methylation may be an important regulatory mechanism underlying the host response to subclinical mastitis and provides a good source for further epigenetic investigation. Together, these findings suggest that subclinical mastitis should be viewed not merely as an infectious condition but as a coordinated epigenetic reprogramming of mammary cellular identity, in which DNA methylation dynamically links immune activation with suppression of lactation-associated functions.

Our dataset's defining feature was the predominance of promoter hypomethylation, which accounted for more than 90% of differentially methylated cytosines. This pattern is consistent with previous reports in bovine mastitis, where global loss of DNA methylation was also observed and linked with transcriptional activation of immune-related pathways [24, 31, 39]. Such convergence across independent studies strengthens the view that hypomethylation is not a random byproduct of infection but a characteristic epigenetic signature of mastitis. Interestingly, similar trends of widespread DNA hypomethylation have also been described in chronic inflammatory disorders in humans, suggesting that sustained inflammation may generally promote erosion of methylation marks [31, 40, 41]. While the precise mechanisms may differ, these parallels highlight that promoter hypomethylation could represent a common strategy by which host tissues maintain prolonged immune activation.

Epigenetic changes in the bovine genome were concentrated at specific hotspots on chromosomes 18, 19, and 11. These regions, largely hypomethylated (sometimes over 90%), indicate a coordinated activation of immune-related genes.

The functional breadth of genes affected by this targeted hypomethylation points to a systemic physiological reset rather than a localized immune effect. KEGG pathway analysis revealed enrichment not only in canonical immune defense pathways such as T- and B-cell receptor signaling, but also across major intracellular signaling cascades, including MAPK, Wnt, mTOR, and FoxO, alongside key endocrine axes like cortisol, growth hormone, and oxytocin. This suggests that epigenetic priming in subclinical mastitis extends well beyond immediate pathogen defense, preparing cells for coordinated shifts in metabolism, stress adaptation, and intercellular communication.

Our integrative analysis identified 1,407 DMEGs, reflecting extensive epigenetic reprogramming of mammary cellular responses to SCM. A dominant theme was the inverse association between promoter methylation and transcriptional activity, whereby hypomethylation was coupled with gene activation, and hypermethylation aligned with gene repression. This pattern illustrates an epigenetically mediated trade-off in which mammary epithelial and immune cells prioritize defense mechanisms at the expense of milk production and metabolic maintenance. Importantly, comparative analysis with external datasets revealed that these signatures are not unique to our study: 235 DMEGs overlapped with the dataset of Wang et al. [39], 128 with the dataset of Wang et al. [24], and 51 genes were consistently shared across all three studies (Table S9), despite differences in host breed, infecting pathogen, and analytical methodology. This cross-study reproducibility indicates that certain methylation–expression couplings may represent conserved molecular features of SCM pathogenesis.

At the gene level, several key inflammatory effectors exemplify this pattern. The alarmin *S100A8* was strongly hypomethylated in our dataset (–39.5%) and transcriptionally upregulated (+ 2.22 log₂FC), consistent with in [39] (−14.5%; + 1.82 log₂FC). Our qPCR validation further confirmed its induction (+ 1.39 log₂FC). Its paralog *S100A9* exhibited a similar regulatory signature (−23.2%; + 2.51 in our data; −14.5%; + 1.25 in [39]), reinforcing the role of the S100 family in neutrophil chemotaxis and amplification of inflammatory responses [42]. The inflammasome component *CASP1* showed parallel findings [43]: hypomethylation and upregulation in both our study (−35%; + 2.25) and in [39] (−14%; + 1.86), supported by qPCR validation (+ 2.38). Likewise, the chemokine *CCL3* [41] demonstrated conserved regulation (–39% methylation, + 1.4 log₂FC in our study; −13%, + 1.9 log₂FC in [39]), contributing to leukocyte recruitment and inflammatory amplification. Beyond the annotated immune mediators, we also identified several uncharacterized loci, including *LOC78974*, *LOC100336589*, and *LOC407171,* which were consistently hypomethylated and showed modest transcriptional induction across datasets. Though these genes are not well studied, their consistent regulatory shifts across independent cohorts suggest they could have undiscovered roles in mammary gland defense against infection. This highlights the need for targeted functional studies to better understand how less-characterized genomic regions affect epigenetic responses to subclinical mastitis and host immunity.

The functional coherence of these findings was reinforced by enrichment analyses. KEGG pathways and GO terms associated with upregulated DMEGs highlighted chemokine signaling, Toll-like receptor signaling, Fc receptor-mediated phagocytosis, NETosis, and antigen processing and presentation. Promoter hypomethylation of chemokines (*CCL2, CCL4, CXCL8, CXCL10)* and their receptors (e.g., *CCR1, CXCR2*) corresponded with heightened capacity for immune cell chemotaxis and recruitment [44]. Upregulation of *TLR4* and its co-receptor *LY96* emphasized enhanced pathogen sensing [45, 46], while activation of the *TREM1–TYROBP–Fc* receptor axis suggested amplification of innate immune signaling. Genes such as *NCF1, MPO*, and histone clusters H2A/H3/H4 pointed to active deployment of antimicrobial mechanisms, including oxidative burst and NET formation [47]. Adaptive immunity was also represented, with MHC class I/II molecules (*BOLA-DMB, DQA5, DRB3, MR1*) [48] and antigen-processing machinery (*CD74, ERAP2)* induced, signaling enhanced antigen presentation and T-cell activation. These findings collectively highlight how promoter hypomethylation primes both innate and adaptive immunity during SCM.

In parallel, promoter hypermethylation was consistently linked to the suppression of lactational and metabolic pathways. Among repressed lactation-associated genes, we focused on *CSN3* as a biologically central and phenotypically informative marker of milk protein suppression. The canonical milk protein gene *CSN3* (κ-casein) [17] provided the most striking example, showing + 24% promoter methylation and strong repression (−5.12 log₂FC in RNA-Seq) in our dataset, closely mirrored in the study by Wang et al. [39] (+ 20%; –5.41 log₂FC). qPCR validation confirmed this downregulation (–9.6 log₂FC; *P* = 0.01125), providing robust evidence that epigenetic silencing of milk protein genes is a conserved mechanism during mastitis.

Additional repressed genes included *PCBD1* (+ 15.9% methylation; −2.11 log₂FC in our data; + 11%; −2.00 in [39]), a cofactor in tetrahydrobiopterin biosynthesis [49], and *OXCT1*, involved in ketone body metabolism [50], further supporting the suppression of biosynthetic and energy-conserving pathways. Regulatory proteins such as PRKAR2B, a cAMP-dependent kinase subunit [51], and DNAJB2, a chaperone involved in ER protein folding, were also hypermethylated and downregulated, indicating downscaling of proteostasis and intracellular signaling capacity [52]. Together, these findings suggest that promoter hypermethylation directs a systematic withdrawal from lactation, metabolism, and protein synthesis, diverting cellular resources toward immune defense.

Our dataset also identified epigenetic regulators as DMEGs, pointing to a deeper layer of transcriptional reprogramming. Genes such as *GADD45A, EXOSC8, EYA2, PRKAA2*, and *SMARCD3* (Table S10) exhibited differential methylation and expression, alongside repression of chromatin stabilizers (*KANSL1L*, Chromobox-like, SOX-like loci) [53] and induction of small RNAs such as *MIR29C*, known to target *DNMT3A/3B.* These changes suggest that the mammary gland not only undergoes epigenetic remodeling but also modulates the machinery responsible for sustaining and reinforcing these changes.

High-throughput sequencing and qPCR validation consistently confirmed the results. Upregulation of *S100A8* (+ 1.39 log₂FC) and *CASP1* (+ 2.38 log₂FC) was experimentally validated, matching methylation data and external datasets. Significant downregulation was observed for *CSN3* (*P* ≤ 0.01125), *PRKAR2B* (*P* ≤ 0.002994), and suppression was also seen in *OXCT1, DNAJB2*, and *PCBD1,* supporting that promoter hypermethylation leads to reduced function in lactational and metabolic genes.

Taken together, these findings position SCM as a methylation-driven reprogramming of mammary epithelial identity, balancing defense activation with lactational suppression. The reproducibility of key gene-level and pathway-level signatures across multiple datasets, pathogens, and breeds underscores their biological importance. While additional validation in larger, pathogen-stratified cohorts will be necessary, the consistent coupling of promoter methylation changes with transcriptional outcomes suggests that milk-cell methylation profiles hold promise for advancing our mechanistic understanding of SCM and could, in the future, contribute to the development of molecular tools for disease monitoring and management. This integrative evidence supports a model in which DNA methylation functions as a regulatory switch linking immune signaling pathways with metabolic and lactational programs, enabling the mammary gland to dynamically redirect cellular resources toward host defense during subclinical mastitis.

An important consideration in interpreting these findings is the relatively small number of biological replicates used for the multi-omics analyses. Although high-throughput sequencing studies often employ limited sample numbers due to cost and analytical complexity, smaller cohorts may reduce statistical power and increase the risk of false discoveries. To mitigate these risks, we applied stringent analytical criteria, including minimum sequencing coverage (≥ 7 × per sample), effect-size filtering for differential methylation (≥ 20% methylation difference), false discovery rate correction (*q-*value < 0.05), and integrative filtering requiring concordant methylation–expression changes. In addition, key candidate genes were experimentally validated using qRT-PCR. The convergence of evidence across independent molecular layers therefore increases confidence in the biological relevance of the identified regulatory signals.

Despite the strong concordance between methylation changes, transcriptional responses, and experimental validation observed in this study, limitations should be considered when interpreting these findings. We acknowledge several limitations of this study and propose directions to address them in future research. The modest sample size inevitably reduces statistical power and may constrain the generalizability of the findings. Although the sequencing depth and analytical framework applied here are consistent with methodological standards for whole-genome bisulfite sequencing studies [54], and comparable genome-wide methylation and transcriptomic investigations in livestock disease models have employed similar group sizes [55–57]. Replicating this work in a larger cohort that incorporates additional healthy controls, diverse breeds, and varying lactation stages will strengthen the robustness and reproducibility of the observed signatures. Another important consideration is the reliance on bulk milk somatic cells as the biological sample. This heterogeneous population, comprising neutrophils, macrophages, lymphocytes, and exfoliated epithelial cells, inevitably masks the distinct epigenetic and transcriptional profiles of individual cell types. At the same time, milk somatic cells remain the most practical and widely accepted choice in mastitis research, as they provide a non-invasive approach and have been consistently used in most previous studies [20, 58–61]. The integrated molecular profile effectively captures overall host response and is ideal for large-scale studies in production animals. However, bulk analysis averages out cell-type-specific molecular differences, possibly obscuring important events. Employing single-cell, quarter-matched, or spatial multi-omics could address this heterogeneity and yield more detailed insights. While this study combined DNA methylation and mRNA data, the lack of other omic layers, such as non-coding RNAs, proteomics, and metabolomics, limited the regulatory overview. Including these aspects in future research will offer a more comprehensive understanding of mastitis biology [62]. We also recognize the importance of functional assays to bridge the gap between association and causality. Locus-specific bisulfite validation, CRISPR-based epigenome editing, and infection models using bovine mammary epithelial cells or organoids could directly test whether methylation changes are causal drivers of the transcriptional outcomes we observed [63]. Despite these challenges, our study provides valuable insights into the genomic and epigenomic signatures of subclinical mastitis in milk somatic cells and establishes a foundation for more extensive investigations. Addressing these limitations through larger, multi-layered, and functionally validated studies will advance understanding of the regulatory mechanisms underlying subclinical mastitis and facilitate their translation into dairy cattle health management.

In summary, this study provides a comprehensive and integrative view of the regulatory networks underlying bovine subclinical mastitis by combining genome-wide DNA methylation with transcriptome profiling in milk somatic cells. We identified key biological processes and functional pathways shaped by epigenetic signatures, offering mechanistic insights into the pathogenesis of SCM. The analysis revealed abundant alterations across the methylome and transcriptome, highlighting five principal axes of response: activation of innate and adaptive immune signaling, metabolic reprogramming toward immune support, remodeling of structural and extracellular pathways, suppression of lactation and hormonal programs, and internal rewiring of transcriptional and epigenetic regulators. Importantly, comparative integration with other mastitis datasets demonstrated a high degree of overlap, pointing to conserved molecular features that transcend pathogen type, breed background, and methodology. Within this core set, several genes, including *CSN3, S100A8, S100A9, CASP1*, and *CCL3,* showed reproducible patterns of promoter methylation changes and transcriptional outcomes, further supported by qPCR validation, underscoring their central role in the epigenetic regulation of mastitis. Although further validation in larger, pathogen-stratified cohorts is needed, these reproducible signals suggest that consistent methylation–expression couplings may hold promise as candidate biomarkers for future mastitis monitoring and control strategies. Ultimately, the regulatory mechanisms revealed here contribute to a deeper understanding of the molecular basis of subclinical mastitis and provide a foundation for developing improved strategies for disease management with potential economic benefits for the dairy industry.

## Conclusion

In summary, this study provides an integrative view of the molecular regulatory networks underlying bovine subclinical mastitis by combining genome-wide DNA methylation profiling with transcriptome analysis in milk somatic cells. We identified key biological processes and functional pathways that define the disease state. The breadth of altered signatures highlights the complexity of subclinical mastitis and underscores the role of epigenetic regulation in shaping host responses.

Our findings indicate that the molecular response to subclinical mastitis is characterized by promoter hypomethylation linked with the activation of immune pathways, metabolic reprogramming that supports inflammatory demands, and promoter hypermethylation associated with suppression of lactation, hormonal signaling, and proteostatic functions. Together, these findings support an epigenetically coordinated immune–lactation trade-off in which mammary epithelial and immune cells prioritize host defense at the expense of milk synthesis during subclinical mastitis.

At the genome-wide level, we identified extensive differentially methylated cytosines, regions, and genes, many of which showed the expected inverse association between promoter methylation and transcription. Comparisons with other mastitis studies showed broad concordance in the regulatory patterns of key genes, suggesting that certain methylation–expression couplings may represent conserved features of the disease across breeds, pathogens, and study designs.

This work establishes a framework for understanding the epigenetic mechanisms of subclinical mastitis in crossbred cattle. Although validation in larger, pathogen-diverse cohorts remains essential, the reproducibility of these regulatory patterns across independent studies suggests their potential utility in guiding future efforts toward early detection, improved disease management, and the exploration of epigenetic resilience in dairy herds.

## Supplementary Information

Additional file 1: Fig. S1. Bismark alignment summary of WGBS data. Stacked area plot showing the proportion of reads in each category across samples. Values are expressed as percentages of total reads per sample. Fig. S2. Manhattan plot of significant differentially methylated regions (DMRs). Each point represents a DMR, plotted by genomic position across chromosomes (*x*-axis) and statistical significance (−log₁₀ *P*-value, *y*-axis). The red dashed line indicates the significance threshold.

Additional file 2: Table S1A. WGBS read quality and trimming metrics. FastQC average/median read length (bp) and total reads, plus Cutadapt % trimmed for each sample (R1/R2). Table S1B. WGBS alignment statistics and CpG methylation counts. Per-sample alignment metrics (total, aligned, ambiguous reads, no genomic sequence) and cytosine context counts, including total CpGs, methylated CpGs, and unmethylated CpGs, for healthy control and subclinical mastitis cows. Table S1C. RNA-seq read quality metrics (FastQC summary). FastQC outputs for each sample (R1/R2), including GC content (%), average sequence length, percentage of failed modules, and total read counts prior to alignment. Table S1D. RNA-seq alignment and mapping statistics. STAR alignment summary showing read mapping outcomes for each sample, including uniquely mapped, total mapped, multiple/too many loci, and categories of unmapped reads (mismatches, too short, other). Percentages are shown relative to total reads. Table S2. Distribution of differentially methylated cytosines (DMCs) across genomic features. Counts of total, hypomethylated, and hypermethylated DMCs identified in promoters, exons, introns, downstream regions, genes, CpG islands, CpG island shores, and CpG island shelves. Table S3A. Genome-wide list of differentially methylated cytosines (DMCs). Genomic coordinates and statistical details of all identified CpG DMCs. Columns include chromosome, start, end, strand, *P*-value, *q*-value (FDR-adjusted), and methylation difference (SCM–HC). Table S3B. Differentially methylated cytosines annotated with gene IDs. Genomic positions, strand, methylation difference (SCM–HC), and associated gene annotations for CpG DMCs. This table links cytosine-level methylation changes to specific genes for downstream functional interpretation. Table S4. Differentially methylated regions (DMRs) identified using 1-kb sliding windows. List of significant DMRs detected with a 1-kb window and 1-kb step size. Columns report chromosome, start and end positions, strand, *P*-value, *q*-value (FDR-adjusted), and mean methylation difference (SCM–HC). Table S5A. Differentially methylated genes (DMGs). List of all DMGs with DMC count and mean methylation difference. Table S5B. Hypermethylated genes. Genes with significant promoter/body hypermethylation, showing DMC counts, mean methylation difference, and status. Table S5C. Hypomethylated genes. Genes with significant hypomethylation, including DMC counts, mean methylation difference, and status. Table S5D. Genes with high DMC counts. Genes carrying multiple DMCs. Columns indicate (i) genes with ≥ 5 DMCs and (ii) genes with ≥10 DMCs. Table S6A. KEGG pathway enrichment of DMGs (BH-adjusted *P* ≤ 0.05). Complete list of KEGG pathways significantly enriched for differentially methylated genes (DMGs), including pathway ID, description, gene count, enrichment score, and BH-adjusted *P*-value. Table S6B. GO Biological Process terms enriched in DMGs (BH-adjusted *P* ≤ 0.05). Full set of enriched GO biological process categories for DMGs, reporting GO ID, term description, gene count, fold enrichment, and BH-adjusted significance. Table S6C. GO Molecular Function terms enriched in DMGs (BH-adjusted *P* ≤ 0.05). Enriched GO molecular function categories associated with DMGs, with GO ID, description, gene count, enrichment score, and corrected *P*-value. Table S6D. GO Cellular Component terms enriched in DMGs (BH-adjusted *P* ≤ 0.05). Cellular component categories enriched in DMGs, providing GO ID, description, gene count, enrichment score, and BH-adjusted significance values. Table S7A. Genome-wide list of differentially methylated and expressed genes (DMEGs). Complete list of 1,407 DMEGs identified by Gaussian Mixture Model (*P* < 0.001) with promoter/exon/intron methylation change >10% and expression change |log₂FC|> 1. Columns report gene ID, mean methylation and expression values in healthy control and SCM groups, Δ methylation, Δ gene expression, and log₂ fold change. Table S7B. Promoter-associated DMEGs. Genes with significant methylation alterations in promoter regions and corresponding expression changes. Table S7C. Exon-associated DMEGs. Genes showing exon methylation differences linked with significant expression changes. Table S7D. Intron-associated DMEGs. Genes with intron-level methylation differences correlated with transcriptional changes. Table S7E. Gene body–associated DMEGs. Genes carrying gene body methylation differences associated with altered expression. Table S7F. Upregulated DMEGs. Genes exhibiting hypomethylation across regulatory regions with concomitant transcriptional upregulation. Table S7G. Downregulated DMEGs. Genes showing promoter or regional hypermethylation accompanied by reduced transcription. Table S8A. KEGG pathway enrichment of upregulated DMEGs (*q* < 0.05). List of significantly enriched KEGG pathways associated with upregulated DMEGs, including pathway ID, description, number of genes, enrichment score, and adjusted *q*-value. Table S8B. GO Biological Process enrichment of upregulated DMEGs (*q* < 0.05). Enriched biological process terms for upregulated DMEGs, showing GO ID, description, gene count, fold enrichment, and *q*-value. Table S8C. GO Molecular Function enrichment of upregulated DMEGs (*q* < 0.05). Enriched molecular function terms, including GO ID, description, gene count, enrichment statistics, and significance. Table S8D. GO Cellular Component enrichment of upregulated DMEGs (*q* < 0.05). Cellular component terms enriched in upregulated DMEGs, reporting GO ID, description, number of genes, fold enrichment, and adjusted *q*-value. Table S8E. Functional annotation clustering of upregulated DMEGs. Summary of 30 annotation clusters derived from DAVID/clusterProfiler, grouping related KEGG and GO terms enriched in upregulated DMEGs. Each cluster includes term description, enrichment score, gene count, and representative genes. Table S9A. Genes overlapping between this study and [39]. List of 235 differentially methylated and expressed genes (DMEGs) shared with study [39], indicating conserved epigenetic regulation across datasets. Table S9B. Genes overlapping between this study and [24]. List of 128 DMEGs overlapping with study [24], highlighting reproducibility across independent mastitis cohorts. Table S9C. Conserved DMEGs across three independent mastitis studies. List of 51 DMEGs consistently shared across the present dataset, study [39], and study [24]. These represent robust, conserved molecular signatures of mastitis across pathogen type, breed background, and methodology. Table S10. Annotated list of epigenetic regulators identified as DMEGs. This table lists differentially methylated and expressed genes (DMEGs) functioning as epigenetic regulators, including RNA exosome components, chromatin remodelers, histone modifiers, and phosphorylation-associated kinases.

## Data Availability

The datasets generated and/or analyzed during the current study are available from the corresponding author on reasonable request.

## Abbreviations

CGI: CpG island
DMC: Differentially methylated cytosine
DMG: Differentially methylated gene
DMEG: Differentially methylated and expressed gene
DMR: Differentially methylated region
ER: Endoplasmic reticulum
FDR: False discovery rate
GAPDH: Glyceraldehyde-3-phosphate dehydrogenase
GMM: Gaussian mixture model
GO: Gene Ontology
GPCR: G protein-coupled receptor
GWAS: Genome-wide association study
HC: Healthy control
KEGG: Kyoto Encyclopedia of Genes and Genomes
log₂FC: Log₂ fold change
MAPK: Mitogen-activated protein kinase
MHC: Major histocompatibility complex
mTOR: Mechanistic target of rapamycin
NETosis: Neutrophil extracellular trap formation
qPCR: Quantitative polymerase chain reaction
qRT-PCR: Quantitative reverse transcription PCR
RNA-seq: RNA sequencing
SCC: Somatic cell count
SCM: Subclinical mastitis
SCP: Subclinical mastitis-positive
STAR: Spliced Transcripts Alignment to a Reference
TPM: Transcripts per million
TLR: Toll-like receptor
TSS: Transcription start site
WGBS: Whole-genome bisulfite sequencing
