# Correction: Integrated methylome–transcriptome profiling reveals epigenetic regulation of immune activation pathways and *CSN3*-associated lactation repression in bovine subclinical mastitis

**DOI:** 10.1186/s40104-026-01462-3

**Published:** 2026-06-09

**Authors:** Shraddha Dwivedi, Amit Kumar, Ujjwal Kumar De, Anuj Chauhan, Ravi Kant Agrawal, Shivani Khanna, Amritanshu Upadhyay, Ayushi Singh, Prem Chand Devatwal, Triveni Dutt

**Affiliations:** 1 Division of Animal Genetics, ICAR – Indian Veterinary Research Institute, Izatnagar, Bareilly, UP 243122 India; 2 Division of Medicine, Indian Veterinary Research Institute, Izatnagar, Bareilly, UP 243122 India; 3 Division of Livestock Production and Management, Indian Veterinary Research Institute, Izatnagar, Bareilly, UP 243122 India; 4 Division of Livestock Products Technology, Indian Veterinary Research Institute, Izatnagar, Bareilly, UP 243122 India

**Correction: J Animal Sci Biotechnol 17, 88 (2026)**

**https://doi.org/10.1186/s40104-026-01400-3**

Following the publication of original article [1], it is reported that there are typographical errors in Table 1: the qPCR log_2_FC value for gene *OXCT1* needs to be corrected from −9.6 to −5.3; the % Promoter methylation change for gene *OXCT1* needs to be corrected from +12.8% to +12.9%; the % Promoter methylation change for gene *DNAJB2* needs to be corrected from +11% to +10.3%.

This correction does not affect findings/interpretation of the original article.

The original article [1] has been corrected.
