# Supplementary figures and images for: Integrated methylome–transcriptome profiling reveals epigenetic regulation of immune activation pathways and CSN3-associated lactation repression in bovine subclinical mastitis
Source: J Anim Sci Biotechnol. 2026 May 10;17:88. doi: 10.1186/s40104-026-01400-3 (PMC13157666; doi:10.1186/s40104-026-01400-3)

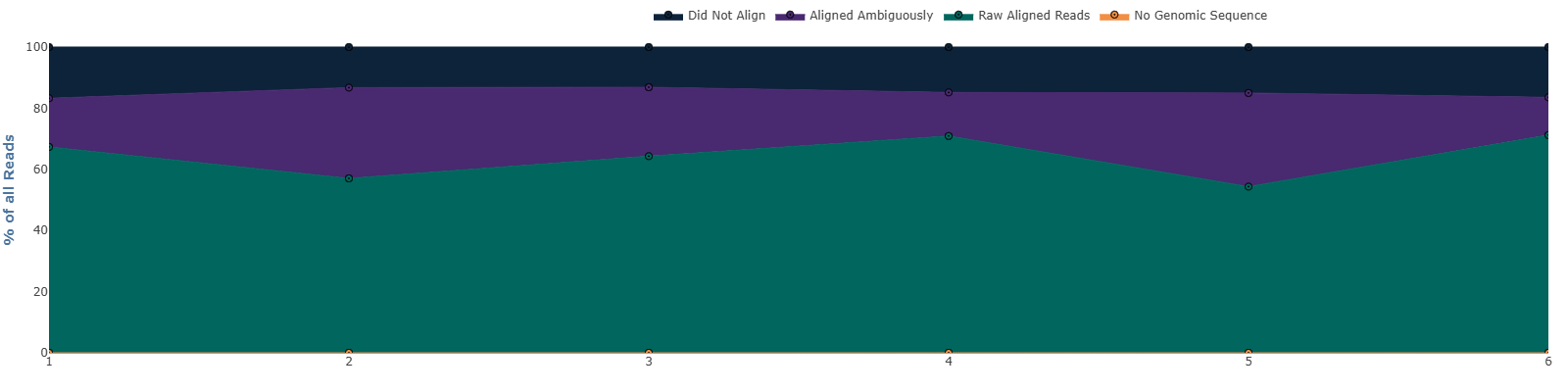

Supplement: Supplementary file 1 — Additional file 1: Fig. S1. Bismark alignment summary of WGBS data. Stacked area plot showing the proportion of reads in each category across samples. Values are expressed as percentages of total reads per sample. Fig. S2. Manhattan plot of significant differentially methylated regions (DMRs). Each point represents a DMR, plotted by genomic position across chromosomes (x-axis) and statistical significance (−log₁₀ P-value, y-axis). The red dashed line indicates the significance threshold. [file 40104_2026_1400_MOESM1_ESM.zip › Additional Figure_S1.png]

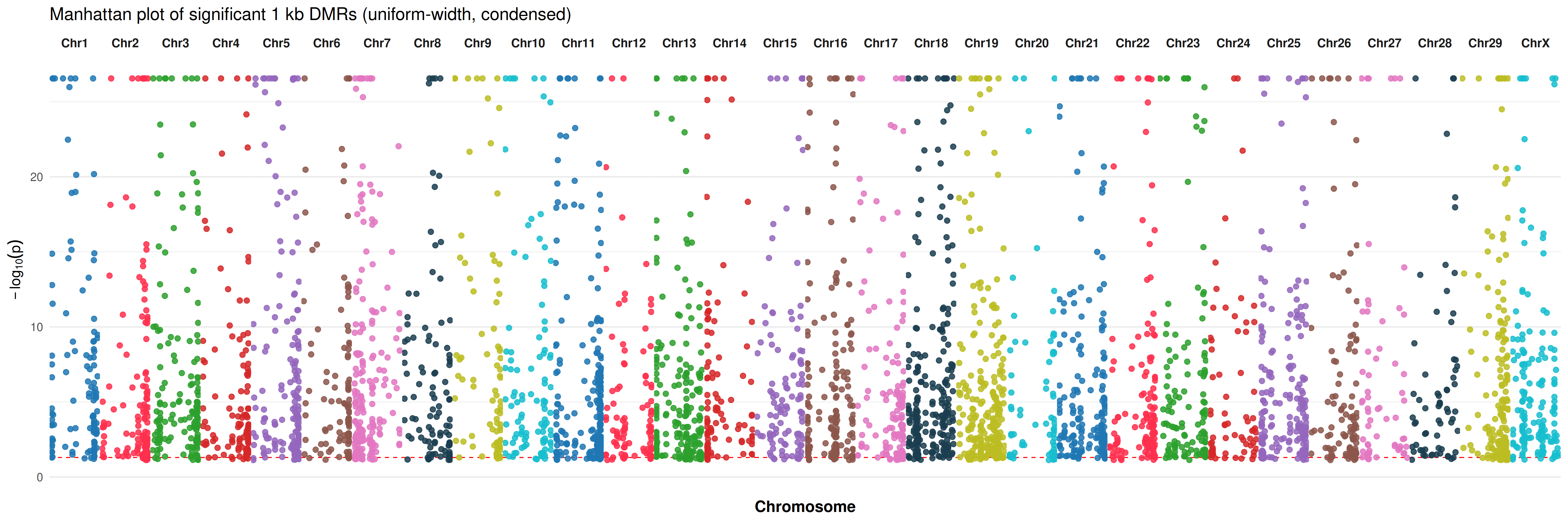

Supplement: Supplementary file 1 — Additional file 1: Fig. S1. Bismark alignment summary of WGBS data. Stacked area plot showing the proportion of reads in each category across samples. Values are expressed as percentages of total reads per sample. Fig. S2. Manhattan plot of significant differentially methylated regions (DMRs). Each point represents a DMR, plotted by genomic position across chromosomes (x-axis) and statistical significance (−log₁₀ P-value, y-axis). The red dashed line indicates the significance threshold. [file 40104_2026_1400_MOESM1_ESM.zip › Additional Figure_S2.png]
